# Theoretical Analysis of Low-Frequency Sound Absorption Owing to the Vibration of Lightweight Powder Using a 1D Beam Model

**DOI:** 10.3390/ma18112611

**Published:** 2025-06-03

**Authors:** Shuichi Sakamoto, Yuya Kawakami, Hiroaki Soeta, Yosuke Kubo

**Affiliations:** 1 Department of Engineering, Niigata University, Ikarashi 2-no-cho 8050, Nishi-ku, Niigata 950-2181, Japan; 2 Graduate School of Science and Technology, Niigata University, Ikarashi 2-no-cho 8050, Nishi-ku, Niigata 950-2181, Japan

**Keywords:** sound absorption coefficient, lightweight powder, longitudinal vibration, 1D beam model, transfer matrix

## Abstract

Lightweight powder-based sound-absorbing materials are characterized by sound absorption peaks at lower frequencies compared to other sound absorption materials of the same thickness. This behavior is attributed to the excitation of longitudinal vibration modes in the powder particles by incident sound waves, wherein acoustic energy is converted into kinetic energy and subsequently dissipated through interparticle interactions. These lightweight, fine powders are artificially engineered acoustic materials. Despite their structural simplicity, they exhibit emergent and complex sound absorption behaviors through fundamental vibrational mechanisms. Representing the powder layer with a transfer matrix simplifies model-based development and enhances versatility as an acoustic element. The powder layer was modeled as a longitudinally oscillating 1D beam, and transfer matrix of the powder layer was derived. To verify the obtained transfer matrix, the experimental values were compared with the theoretical values for a single powder layer. In addition, both were compared for the case of other acoustic elements stacked on top of each other, which were close to each other. The theoretical values were compared with the experimental values, which were close to each other.

## 1. Introduction

Lightweight powder-based sound-absorbing materials (SAMs) are characterized by sound absorption peaks at lower frequencies compared to other SAMs of the same thickness. Therefore, powder-based SAMs are considered enablers of smaller, lighter SAM designs.

Lightweight powders with particle sizes of several tens of micrometers exhibit sound absorption peaks at lower frequencies than other porous SAMs. In such powders, boundary layer viscosity—typically the dominant absorption mechanism in porous materials—has minimal influence. Instead, acoustic energy is attenuated through interparticle interactions induced by longitudinal vibrations of the powder particles, excited by incident sound waves [1]. These lightweight, fine powders are artificially engineered acoustic materials. Despite their structural simplicity, they exhibit emergent and complex sound absorption behaviors through fundamental vibrational mechanisms [2].

The lightweight powders analyzed in this study have bulk densities in the range of 13.9–82 kg/m^3^, comparable to that of conventional sound-absorbing materials such as glass wool, which typically ranges from 10 to 96 kg/m^3^. At a layer thickness of 20 mm, the sound absorption peak of glass wool occurs around 2000 Hz, while that of the lightweight powders lies between 500 and 1000 Hz. Therefore, these powders demonstrate superior absorption in the low-frequency range compared to standard porous materials. Regarding acoustic powder properties, studies exist on the sound absorption properties of powder beds made of binary powder mixtures [3], modal analysis of powder layers as a five-degree-of-freedom forced vibration system and calculation of acoustic impedance [4], and exploration of the sound absorption effect of boundary-layer viscosity beyond sound absorption by vibration [5]. Studies have also examined the conditions under which the sound absorption effect of powder layer vibration appears [6,7] and the vibration isolation and sound insulation properties [8] of silica aerogels. In recent years, the Biot model [9] has been used to study the impact of casing on the sound absorption of fine spherical-granular materials [10].

SAMs are often combined with several other materials to achieve a higher sound absorption effect. The transfer matrix method is a useful theoretical tool for analyzing SAMs combined in this manner. Therefore, representing the powder layer with a transfer matrix simplifies model-based development and enhances versatility as an acoustic element. Previous studies’ theoretical analyses [4,5] have enabled powder layer acoustic impedance determination. However, the absence of transfer matrices prevented theoretical value calculations for combinations with other SAMs. A powder layer transfer matrix was attempted using the two-thickness method [11], but the theoretical values deviated from the experimental values in the high-frequency range.

In this study, the powder layer was modeled as a longitudinally oscillating 1D beam, and transfer matrix of the powder layer was derived. The Biot model [9], commonly used to derive sound absorption coefficients, requires nine experimentally determined parameters. In contrast, the model proposed in this study allows for theoretical analysis based on only three of these nine parameters: bulk density, loss factor, and a newly introduced parameter, the first-order sound absorption peak frequency. This reduction in required parameters represents a significant simplification. The loss factors required in the theoretical analysis were also determined. To verify the obtained transfer matrix, the experimental values were compared with the theoretical values for a single powder layer. In addition, both were compared for the case of other acoustic elements stacked on top of each other. This method is anticipated to be applicable to the design and improvement of acoustic performance in systems such as loudspeaker enclosures incorporating fine powder materials [12].

## 2. Measurement Overview and Preliminary Investigation 

### 2.1. Measurement Apparatus and Samples

**Figure 1:**
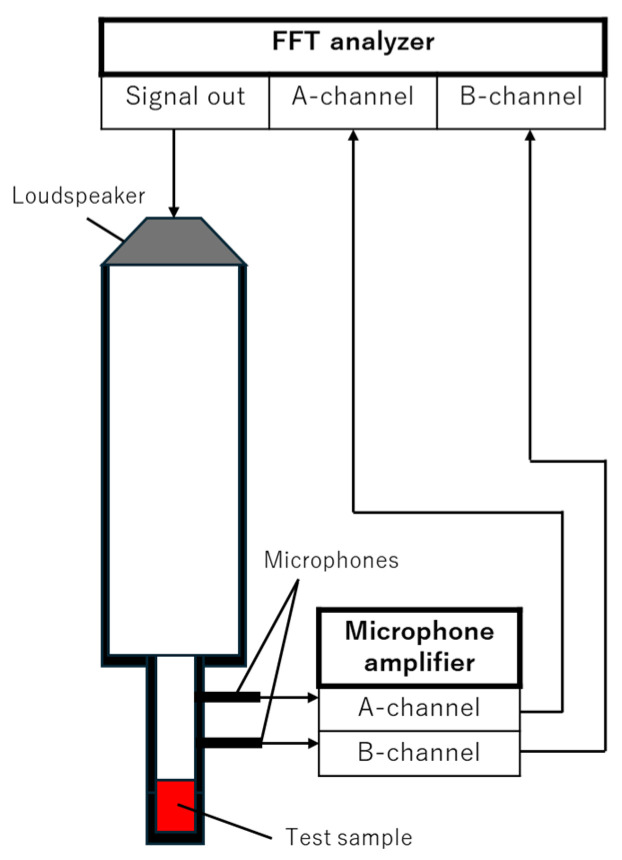
Experimental setup for measuring SACs of powder samples.

Figure 1 shows a schematic of the actual measurement apparatus for measuring the normal incident sound absorption coefficient (SAC). The measurement sample was mounted in a Brüel–Kjær Type 4206 two-microphone impedance-measuring tube (Nærum, Denmark).

The SAC was measured as follows: A sinusoidal signal was output from a signal generator built into an FFT analyzer DS–3000 (Ono Sokki, Kanagawa, Japan). The sound waves can be treated as plane waves because their wavelength is sufficiently smaller than the impedance tube diameter. The transfer function between the sound pressure signals obtained from the two microphones attached to the impedance tube is then measured by the FFT analyzer. The distance between the microphones is 20 mm. The measured transfer function was used to calculate the normal incident SAC according to ISO 10534-2 (2023) [13]. The inner diameter of the acoustic tube determines the frequency limit for the formation of plane waves, and a small tube with an inner diameter of 29 mm was used in this study. The upper frequency limit that can be measured in this case is 6400 Hz according to ISO 10534-2 [13]. The lower frequency limit is 500 Hz, the measurement accuracy of which is guaranteed by the impedance tube specifications. In this study, the experimental results for 100–6400 Hz are described for reference.

The powder controlled in a storage area at a constant temperature (20 °C) and humidity (35%) was filled into an aluminum alloy sample holder (inner diameter 29 mm), and the SAC was measured. The powder was filled by tapping. The powders employed in this study are granular materials. Unlike foam- or fiber-based materials, their volume does not change appreciably under compression. As a result, bulk density remains nearly constant even after tapping, and the corresponding change in sound absorption performance is minimal. Preliminary tests revealed that the sample holder did not affect sound absorption or acoustic properties.

**Figure 2:**
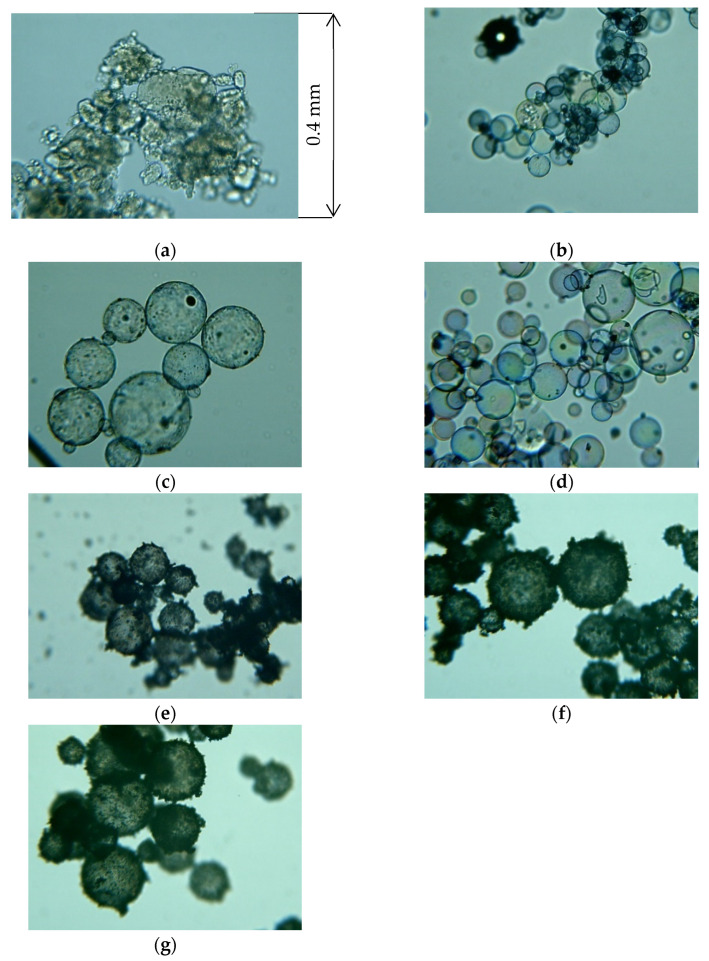
Micrographs of the powder materials: (**a**) granulated silica; (**b**) hollow plastic beads A (Expancel^®^ 920DE40d30); (**c**) hollow plastic beads B (Matsumoto micro sphere^®^ F–80DE); (**d**) hollow glass beads; (**e**) calcium-carbonate-coated hollow plastic beads A (EMC–40(B)); (**f**) calcium-carbonate-coated hollow plastic beads B (EMC–80(B)); (**g**) calcium-carbonate-coated hollow plastic beads C (EMC–120(α)).

**Figure 3:**
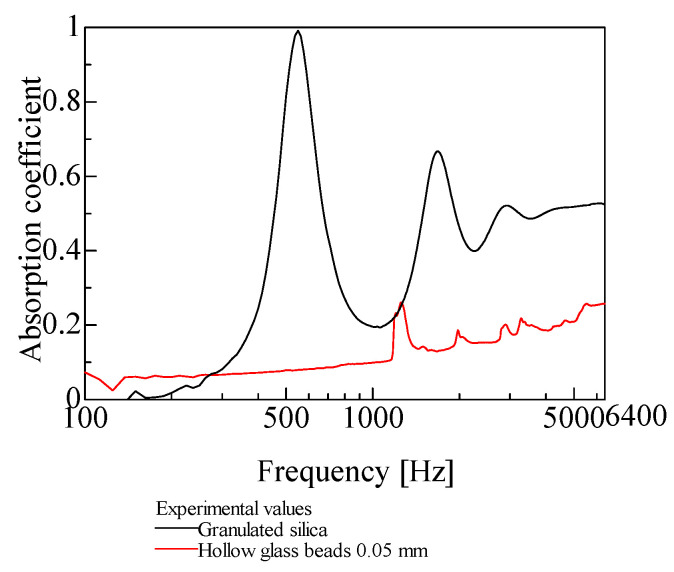
Comparison of sound absorption coefficient between granulated silica and hollow glass beads 0.05 mm.

Figure 2 presents the micrographs of the powder samples used for the measurements, while Table 1 summarizes their characteristics. The powders examined in this study are those with bulk densities classified as exhibiting significant sound absorption owing to longitudinal vibration, as indicated in a previous report [7], consistent with the primary objective of this method. Figure 3 shows the sound absorption coefficients of granulated silica (grain diameter: 0.0428 mm; bulk density: 0.0568 g/cm^3^) and solid glass beads (grain diameter: 0.05 mm; bulk density: 1.45 g/cm^3^). As shown in Figure 3, despite having nearly the same grain size, sound absorption in the heavier powder is dominated by boundary layer viscosity, and minimal absorption occurs in the low-frequency range due to particle vibration. Accordingly, such heavy powders are excluded from the scope of this study.

The particle size represents the average Feret diameter [14] (constant tangential diameter) derived from the micrographs to minimize the influence of particle orientation during measurement. The particle size was not used as a parameter in this study, although it was included as a reference. The first-order peak sound absorption frequencies were obtained from experiments measuring the SAC at a powder layer of 20 mm.

**Figure 4:**
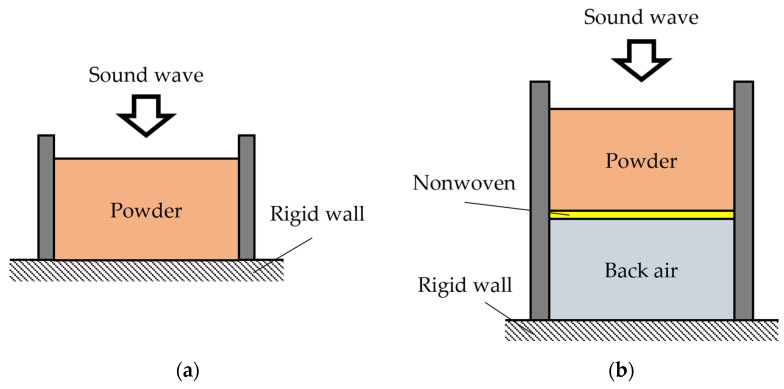
Schematic of the samples: (**a**) powder layer only; (**b**) powder layer with back air space.

Figure 4a shows a schematic diagram of the powder layer alone, and Figure 4b shows a schematic diagram of the powder layer filled on top of the nonwoven fabric, with the nonwoven fabric placed at the upper end of the back air space. The nonwoven fabric used, 3A01A (Toyobo Co., Ltd., Osaka, Japan), is relatively thin and designed with low ventilation resistance to avoid impacting the powder’s acoustic properties. The measured ventilation resistance was 0.34 × 10^3^ Pa-s/m and the thickness was 0.39 mm. The ventilation resistance of the nonwoven fabric was measured using a ventilation resistance tester KES-F8-AP1 (Kato Tech Co., Ltd., Kyoto, Japan).

### 2.2. Demonstration of Sound Absorption Due to Longitudinal Vibration

The preliminary experimental results are presented to demonstrate sound absorption caused by the longitudinal vibration of a powder layer. These results were confirmed by the authors before the start of this study [4]. They are included in the first part of this report to address potential doubts regarding the existence of longitudinal vibrations.

**Figure 5:**
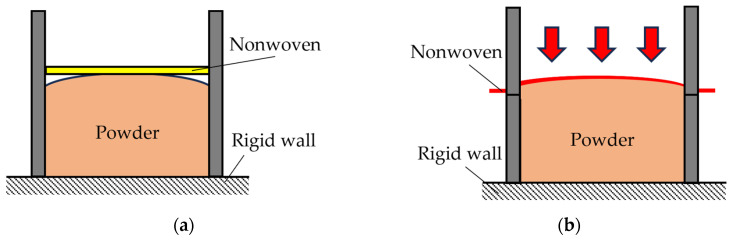
Schematic representation of the samples: (**a**) nonwoven fabric placed on top; (**b**) suppression of longitudinal vibration.

The method for demonstrating that the powder absorbs sound due to longitudinal vibrations is as follows: If the powder is compressed to prevent longitudinal vibration, the sound-absorbing effect should be significantly reduced. To test this, we measured the sound absorption coefficient of a powder layer with a slightly greater volume than the sample tube. The powder was then pressed down using a nonwoven fabric (3A01A) to inhibit longitudinal vibrations. A schematic representation of this setup is shown in Figure 5.

**Figure 6:**
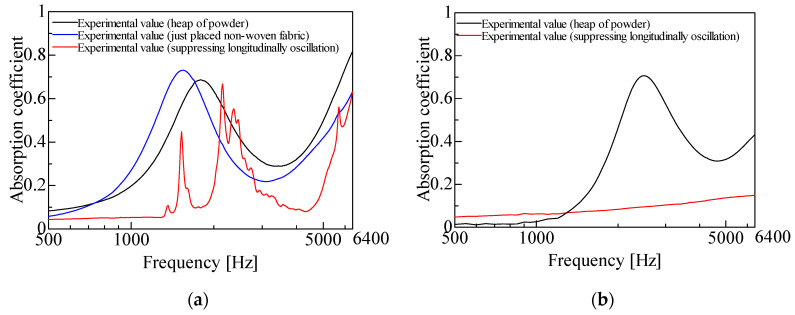
Comparison of experimental values: (**a**) granulated silica, *l_p_* = 5 mm; (**b**) hollow glass beads, *l_p_* = 5 mm.

Figure 6a,b presents the experimental results for two different powders: granulated silica and hollow glass beads. When the powders were pressed down to suppress longitudinal vibrations, their sound absorption performance was significantly reduced. This confirms that the sound absorption properties of lightweight powders are primarily due to their longitudinal vibrations.

One possible concern is that the observed change in sound absorption may be attributed to the acoustic effect of the nonwoven fabric rather than the suppression of longitudinal vibrations. To address this, Figure 6a also includes measurements where the nonwoven fabric was simply placed on top of the powder layer without pressing it down. The results show that this had a negligible effect on sound absorption. However, when the fabric was merely placed on the powder layer, the sound absorption peak frequency slightly decreased, and the peak absorption value slightly increased. This is likely due to the slight increase in the powder layer’s thickness, as mentioned earlier.

## 3. Theoretical Analysis

### 3.1. Transfer Matrix Method

**Figure 7:**
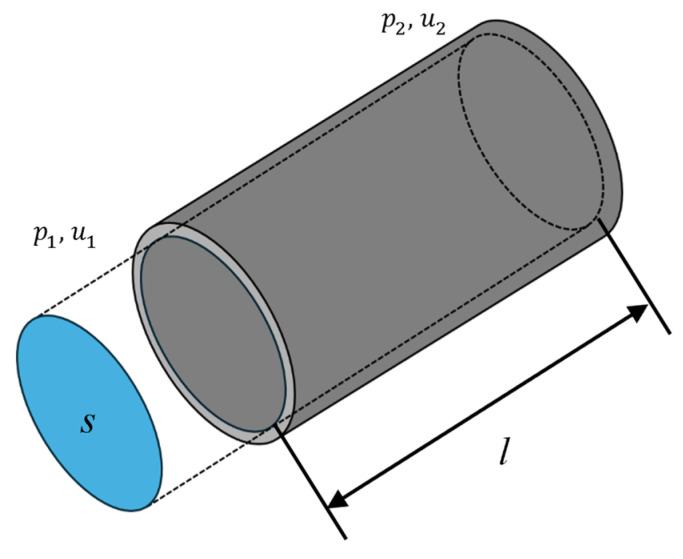
Acoustic element.

In this study, the theoretical SAC values were calculated using the transfer matrix method. For the acoustic element shown in Figure 7, if the sound pressure and particle velocity are $p_{1}$, $p_{2}$ and $u_{1}$, $u_{2}$ for the incident and terminal ends of the acoustic element, respectively, the acoustic element can be expressed using the transfer matrix as follows:

(1) $$\left[\begin{array}{c} p_{1}\\ Su_{1} \end{array}\right]=\left[\begin{array}{cc} A & B\\ C & D \end{array}\right]\left[\begin{array}{c} p_{2}\\ Su_{2} \end{array}\right]$$

Based on the 1D wave equation, the transfer matrix for sound pressure and volume velocity can be expressed as follows:

(2) $$T=\left[\begin{array}{cc} A & B\\ C & D \end{array}\right]=\left[\begin{array}{cc} \cosh(\gamma l) & \frac{Z_{c}}{S}\sinh(\gamma l)\\ \frac{S}{Z_{c}}\sinh(\gamma l) & \cosh(\gamma l) \end{array}\right]$$

where *S* is the cross-sectional area of the acoustic element, *l* is the length, *γ* is the propagation constant, and *Z_c_* is the characteristic impedance. Therefore, to obtain the transfer matrix of the acoustic element, *γ* and *Z_c_* are required.

### 3.2. Characteristic Impedance and Propagation Constants of Powder Layers

In previous studies, vibration analysis of longitudinally oscillating beams was conducted using a transfer matrix [15,16,17]. In the present study, the powder layer was used as a 1D beam model to derive the transfer matrix. In other research domains, analytical models have been extended to two- and three-dimensional representations [18]. However, in acoustic engineering, such extensions are typically considered too complex for analytical approaches and are usually addressed using commercial numerical simulation software (Comsol Multiphysics® Version 5.2). In contrast, the present study seeks a simplified theoretical model that can clearly capture the underlying physical mechanisms.

**Figure 8:**
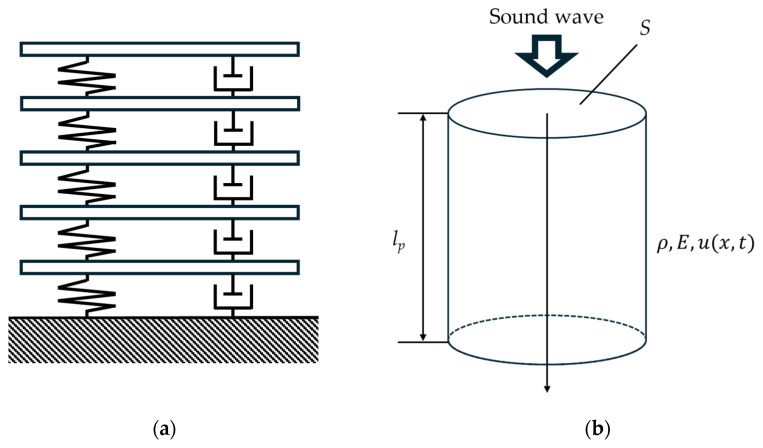
Analytical models: (**a**) 5-DOF; (**b**) longitudinally vibrating 1D beam model.

The powder layer was subjected to incident plane waves normal to the sample surface. Therefore, only powder layer longitudinal vibrations along the tube’s longitudinal direction are assumed to occur, with no radial vibrations generated. The peak sound absorption frequencies of the powder layer increase threefold from the first to the second order and fivefold from the first to the third order. This is similar to the vibration modes of a longitudinally vibrating 1D beam with one fixed end. Therefore, the propagation of sound waves in the powder layer is modeled as a longitudinally vibrating 1D beam. Figure 8a presents the five degrees of freedom (5-DOF) model used in previous work, while Figure 8b shows the proposed longitudinally vibrating 1D beam model.

If the displacement *u* of the beam is a function of the position *x* on the *x*-axis and time *t*, *u*(*x*, *t*), the wave equation is obtained as follows:

(3) $$\rho \;\frac{\partial^{2}u}{\partial t^{2}}=E\frac{\partial^{2}u}{\partial x^{2}}$$

where *ρ* is the beam density, which is equal to the powder layer bulk density. *E* is the Young’s modulus of the beam (=powder layer).

The velocity *C* of the longitudinal elastic wave propagating in the model is given by Equation (4).

(4) $$C=\sqrt{\frac{E}{\rho }}$$

The first-order eigenfrequency occurs at a frequency such that four times the thickness of the powder layer corresponds to the wavelength. From the relationship between the speed of sound in the medium, frequency, and wavelength, the following equation is obtained:

(5) $$f_{p1}=\frac{C}{4l_{p}}$$

From Equations (4) and (5), Equation (6) is obtained for Young’s modulus of the powder layer.

(6) $$E={\rho \left(4f_{p1}l_{p}\right)}^{2}$$

The governing equation for powder layer vibration is the wave equation, which is the same as that used for the propagation of sound waves in air. Therefore, as in Equation (2), for a 1D beam mode, the transfer matrix *T_p_* for the pressure and volume velocity at both ends of the beam can be established. The transfer matrix *T_p_* for the powder layer is derived using expressions for the propagation constant and characteristic impedance of the surrounding air layer.

(7) $$T_{p}=\left[\begin{array}{cc} \cosh(\gamma_{p}l_{p}) & \frac{Z_{p}}{S}\sinh(\gamma_{p}l_{p})\\ \frac{S}{Z_{p}}\sinh(\gamma_{p}l_{p}) & \cosh(\gamma_{p}l_{p}) \end{array}\right]$$

(8) $$\gamma_{p}=\rho C=j\omega \sqrt{\frac{\rho }{E}}$$

(9) $$Z_{p}=\frac{i\omega }{C}=\sqrt{\rho E}$$

This takes the same form as the transfer matrix of the acoustic elements in Equation (2). Thus, *Z_p_* and *γ_p_* can be treated as the characteristic impedance and propagation constants of the powder layer, respectively.

Equation (7) represents the transfer matrix of the beam without attenuation. Here, energy dissipation occurs in the powder layer due to inter-particle interactions. Therefore, Young’s modulus *E* of the powder obtained in Equation (6) is expressed using the loss factor η as follows:

(10) $$E^{*}=E\left(1+i\eta \right)$$

E^*^ is the complex Young’s modulus, where the imaginary part represents damping; The loss factor *η* is a constant. By substituting E^*^ into *E* in Equations (8) and (9), the transfer matrix of the beam with damping can be obtained. The loss factor is defined as the value that achieves the best agreement between theoretical and experimental results at the first-order peak frequency, with details provided in the next section.

### 3.3. Characteristic Impedance and Propagation Constants of Nonwoven Fabrics

The Delany–Bazlry [19], Miki [20], and Komatsu [21] models are models of the airborne sound properties of fabric materials.

In this study, the Miki model [20] was used to calculate the characteristic impedance and propagation constants. The flow resistivity σ of the nonwoven fabric was obtained using Equation (11).

(11) $$\sigma =\frac{R_{non}}{l_{non}}$$

where *R_non_* is the nonwoven fabric ventilation resistance and *l_non_* is its thickness.

By substituting the flow resistivity *σ* into the following equation, the propagation constants *γ_non_* and characteristic impedance *Z_non_* of the nonwoven fabric are obtained, where *f* is the frequency.

(12) $$Z_{non}=\left(1+0.0699{\left(\frac{f}{\sigma }\right)}^{-0.632}\right)-j\left(-0.107{\left(\frac{f}{\sigma }\right)}^{-0.632}\right)$$

(13) $$\gamma_{non}=\left(\left(\frac{\omega }{c_{0}}\right)\left(0.160{\left(\frac{f}{\sigma }\right)}^{-0.618}\right)\right)+j\left(\left(\frac{\omega }{c_{0}}\right)\left(1+0.109{\left(\frac{f}{\sigma }\right)}^{-0.618}\right)\right)$$

### 3.4. Characteristic Impedance and Propagation Constants of the Air Space

The characteristic impedance and propagation constants *Z*_0_ and *γ*_0_ of the air layer can be expressed in terms of $\rho_{0}c_{0}$ and wavenumber, respectively, neglecting attenuation, as follows:

(14) $$Z_{0}=\rho_{0}c_{0}$$

(15) $$\gamma_{0}=\frac{i\omega }{c}$$

### 3.5. Cascade Connection of Four-Terminal Networks in Equivalent Circuits

Having obtained the characteristic impedance and propagation constants for the powder, nonwoven, and air layers in Section 3.1, Section 3.2, Section 3.3 and Section 3.4, they can be substituted into Equation (1) to obtain the respective transfer matrices. Let *T_p_*, *T_non_*, and *T_ba_* be the transfer matrices of the powder, nonwoven, and air layers, respectively.

**Figure 9:**
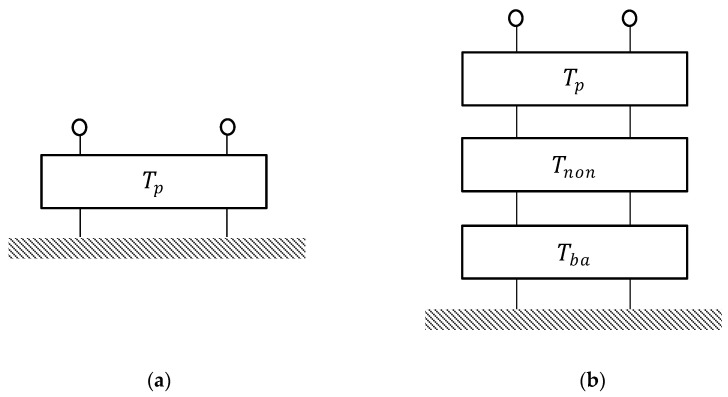
Equivalent circuit of the samples: (**a**) powder layer only; (**b**) powder layer with back air space.

Figure 9a,b shows the equivalent circuits corresponding to Figure 4a,b, respectively. When the four-terminal networks corresponding to several acoustic elements are cascaded, as shown in Figure 9b, the overall transfer matrix *T_all_* is obtained by multiplying the transfer matrices of the respective acoustic elements. In other words, the transfer matrix *T_all_* of the entire acoustic system shown in Figure 4b can be expressed as follows:

(16) $$T_{all}=T_{p}\times T_{non}\times T_{ba}$$

### 3.6. SAC Calculation

For the transfer matrix of the acoustic elements shown in Equation (1), the termination of the sample is a rigid wall; so, the particle velocity *u*_2_ = 0 can be assumed to obtain Equation (17).

(17) $$\left[\begin{array}{c} p_{1}\\ Su_{1} \end{array}\right]=\left[\begin{array}{c} Ap_{2}\\ Cp_{2} \end{array}\right]$$

The specific acoustic impedance *z*_0_ of the entire sample viewed internally from the sample incident surface is given as follows:

(18) $$z_{0}=\frac{p_{1}}{u_{1}}=\frac{A}{C}S$$

where the relationship between the specific acoustic impedance *z*_0_ and the complex reflectance *R* is expressed in Equation (19).

(19) $$R=\frac{z_{0}-\rho_{0}c_{0}}{z_{0}+\rho_{0}c_{0}}$$

The overall SAC, *α*, is expressed by the relationship between the SAC and reflection coefficient *R*, as shown in Equation (20).

(20) $$\alpha =1-{\left|R\right|}^{2}$$

### 3.7. Determination of the Loss Factor

**Figure 10:**
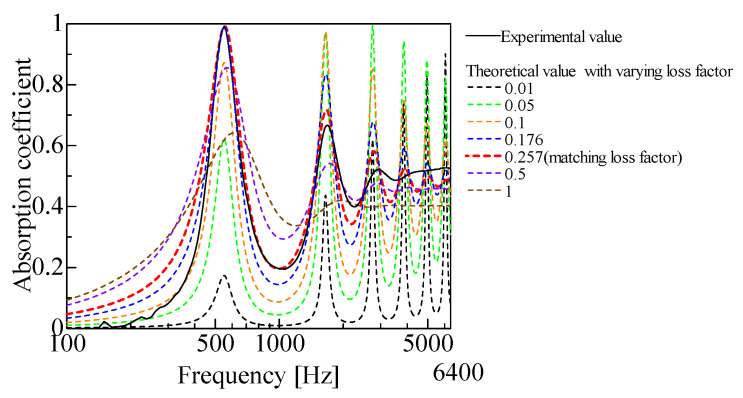
Sensitivity analysis of loss factor. (granulated silica).

**Figure 11:**
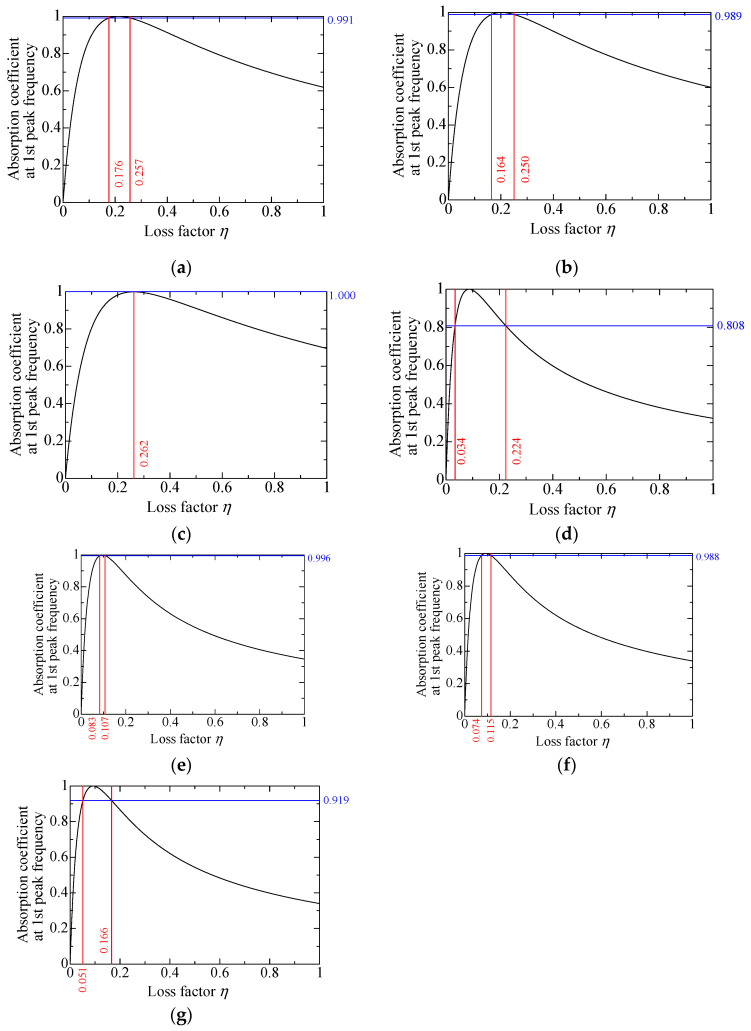
Estimation of loss coefficients consistent with experimental values at the first absorption peak: (**a**) granulated silica; (**b**) hollow plastic beads A (Expancel^®^ 920DE40d30); (**c**) hollow plastic beads B (Matsumoto micro sphere^®^ F–80DE); (**d**) hollow glass beads; (**e**) calcium-carbonate-coated hollow plastic beads A (EMC–40(B)); (**f**) calcium-carbonate-coated hollow plastic beads B (EMC–80(B)); (**g**) calcium-carbonate-coated hollow plastic beads C (EMC–120(α)).

To calculate the propagation constant and characteristic impedance of the powder layer, as described in Section 3.2, determining the loss factor *η* is necessary. As the loss factor changes, the SAC at the primary peak frequency also changes. Therefore, the loss factor with the best agreement between the experimental and theoretical values is searched for when the loss factor varies from 0 to 1. Figure 10 presents the sensitivity analysis of the loss factor for granulated silica. The first-order peak of the sound absorption coefficient initially increases with the loss factor but gradually decreases beyond a certain point. A loss factor of 0.257 was selected for granulated silica, as it yielded the best agreement between theoretical and experimental sound absorption curves. The theoretical SAC values were calculated according to Section 3.6. Figure 11 shows the relationship between the loss factor and first-order peak SAC for each of the powders listed in Table 1. For granulated silica, hollow plastic beads A, hollow glass beads, and calcium-carbonate-coated hollow plastic beads A, B and C, two loss factors were obtained, for which the experimental and theoretical SACs matched. For hollow plastic beads B, only one loss factor was obtained. Table 2 shows the loss factors obtained for each powder, listed from the lower to the higher *η*_1_ and *η*_2_. The respective loss factors are verified in Section 4.

## 4. Verification of the Transfer Matrix of the Powder Layer

### 4.1. Comparison of the Experimental and Theoretical Values of the SAC in the Powder Layer Alone

**Figure 12:**
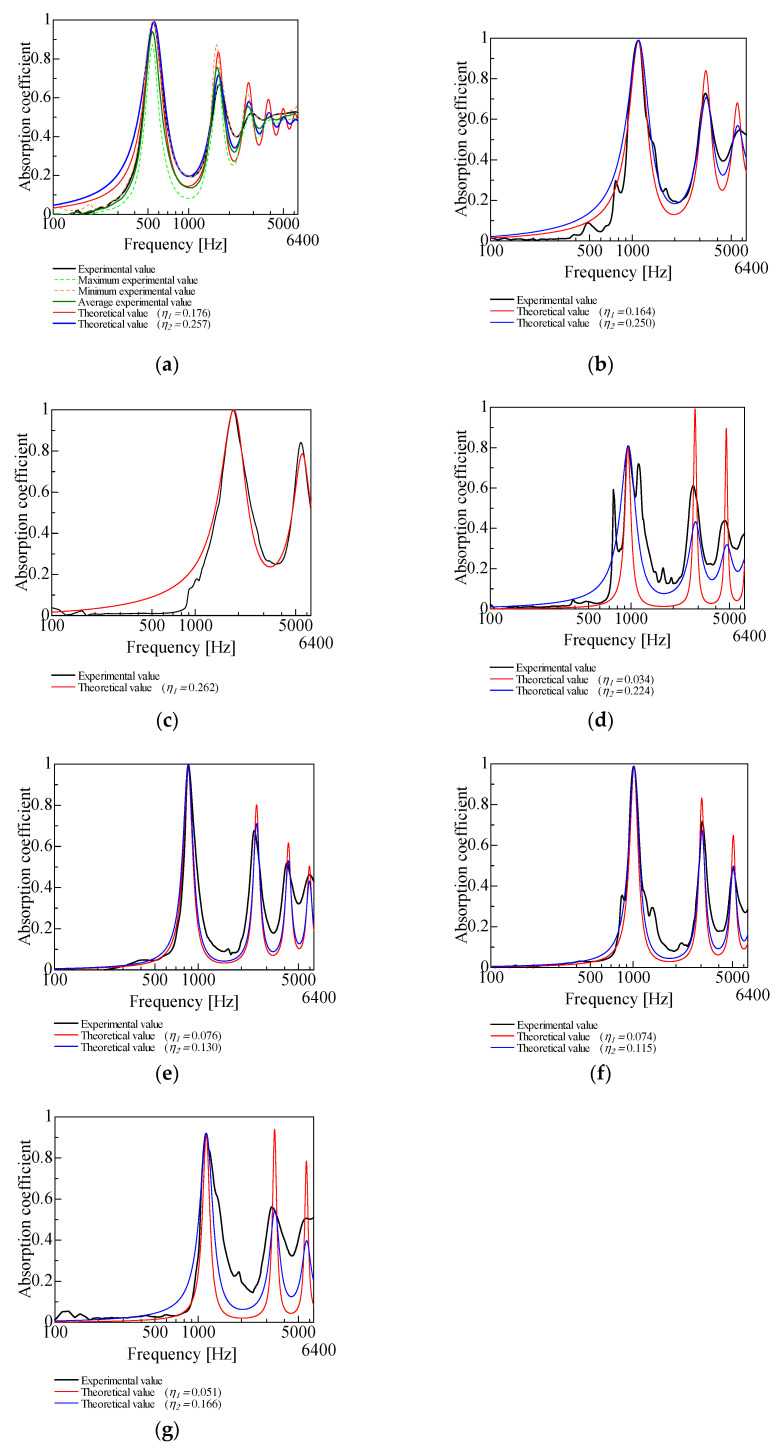
Comparison of experimental and theoretical values (*l_p_* = 20 mm): (**a**) granulated silica; (**b**) hollow plastic beads A (Expancel^®^ 920DE40d30); (**c**) hollow plastic beads B (Matsumoto micro sphere^®^ F–80DE); (**d**) hollow glass beads; (**e**) calcium-carbonate-coated hollow plastic beads A (EMC–40(B)); (**f**) calcium-carbonate-coated hollow plastic beads B (EMC–80(B)); (**g**) calcium-carbonate-coated hollow plastic beads C (EMC–120(α)).

In the transfer matrix of the powder obtained in Section 3, the validity of the transfer matrix was verified by multiplying the transfer matrix with other acoustic elements and comparing the experimental and theoretical values. Figure 12 shows the experimental and theoretical values of the SAC of the powder layer alone, as shown in Figure 4a. The powder layer thickness was 20 mm. In Figure 12a, the maximum, minimum, and mean values from 10 experimental trials with granulated silica are shown to validate the selection of the theoretical values. Near the first-order absorption peak, the theoretical curves align closely with the experimental upper and lower bounds, indicating that the model achieves a high level of accuracy. Theoretical values using the respective loss factors are given for granulated silica, hollow plastic beads A, hollow glass beads, and calcium-carbonate-coated hollow plastic beads A, B and C for which two loss factors were obtained. Figure 12a,b,d–g shows that the loss factors that tend to be closer to the experimental values are all *η*_2_, based on the approximate shape of the sound absorption curves. In Figure 12c, only *η*_1_ was determined. In both comparisons, the theoretical values for the powder layer alone reproduce the experimental values well if the appropriate loss factor is chosen. For this reason, these loss factors were used in the subsequent theoretical analysis.

Table 3 shows the percentage error of the first peak sound absorption frequency of the theoretical values compared to the experimental values. For all powders, the error was of less than 1%. This finding suggests that this study’s transfer matrix can enable estimation of the powder layer SAC.

### 4.2. Comparison of Experimental and Theoretical Values for the SAC with a Back Air Space

Verification was conducted to determine whether appropriate theoretical values could be obtained by multiplying the transfer matrices of other acoustic materials with those of the powder layer. Specifically, the case where a back air space is installed by adding a nonwoven fabric as a support layer behind the powder layer, as shown in Figure 4b, is verified. The powder layer was 20 mm, and the air layer behind was 20 mm.

**Figure 13:**
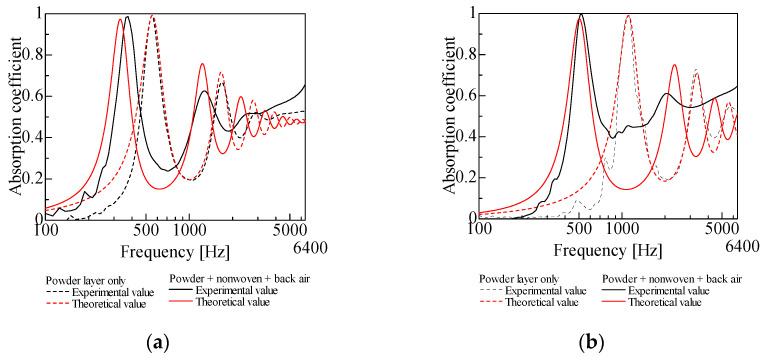

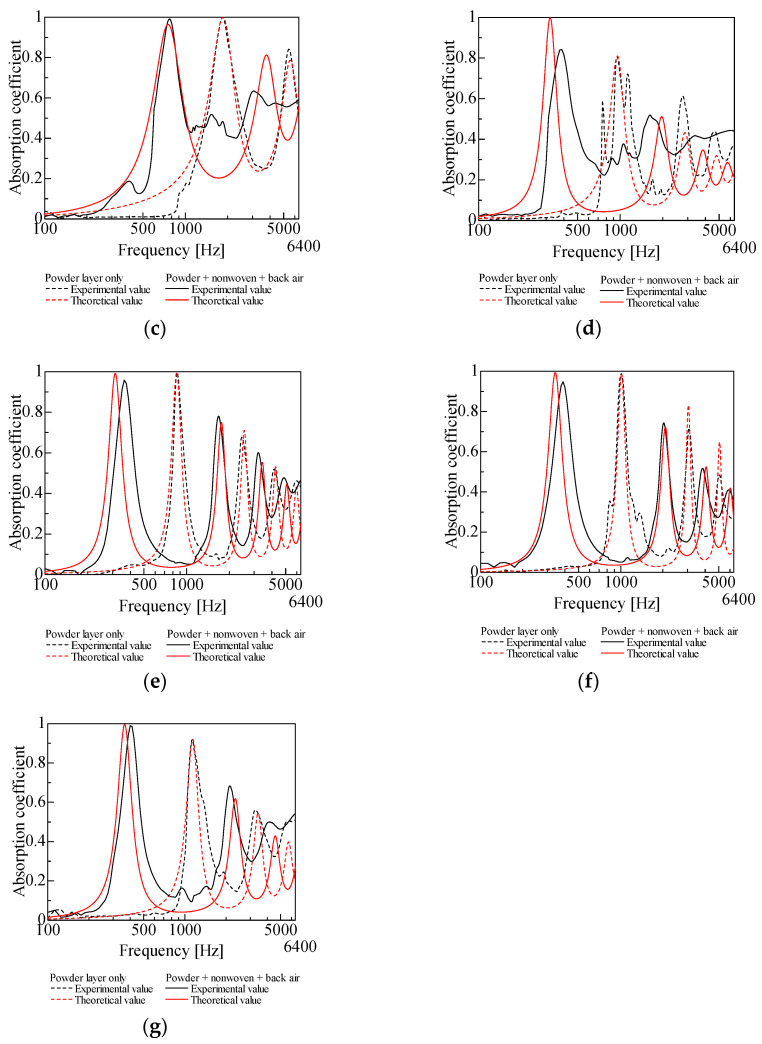
Comparison of experimental and theoretical values (*l_p_* = 20 mm + nonwoven fabric + back air space 20 mm): (**a**) granulated silica; (**b**) hollow plastic beads A (Expancel^®^ 920DE40d30); (**c**) hollow plastic beads B (Matsumoto micro sphere^®^ F–80DE); (**d**) hollow glass beads; (**e**) calcium-carbonate-coated hollow plastic beads A (EMC–40(B)); (**f**) calcium-carbonate-coated hollow plastic beads B (EMC–80(B)); (**g**) calcium-carbonate-coated hollow plastic beads C (EMC–120(α)).

Figure 13 compares the experimental and theoretical values of the SACs. When an air space is installed behind porous material, the sound absorption peak often shifts to lower frequencies due to the increased overall thickness of the sound-absorbing structure. The theoretical values also shifted to the low-frequency side, following the transition of the experimental values to the low-frequency side (see Figure 13). This shows that the theoretical values were successfully obtained by adding the other acoustic elements of the transfer matrix of the powder layer. The present method is applicable to other lightweight powders not examined in this paper, provided they satisfy the conditions required for longitudinal vibration. As the sound absorption mechanism remains the same, such powders fall within the applicable scope of this model [7].

Discrepancies between the theoretical values derived using the 1D beam model and the experimental data are observed at higher frequencies. These deviations are attributed primarily to two factors: first, the loss coefficient assumed in this study is frequency-independent; second, boundary layer viscosity, which becomes more pronounced at higher frequencies, is not considered in the present model. Future improvements may involve incorporating frequency-dependent loss coefficients and extending the model to include boundary layer viscosity effects to enhance predictive accuracy.

Table 4 shows the experimental and theoretical first-peak sound absorption frequencies and the percentage error of the theoretical values relative to the experimental values. For hollow plastic beads A and B and calcium-carbonate-coated hollow plastic beads A and B, the percentage error was of less than 3%, but it exceeded 8% for granulated silica, hollow glass beads, and calcium-carbonate-coated hollow plastic beads C.

### 4.3. Powders to Which This Method Can Be Applied

The 1D beam model used in this study is intended for theoretical analysis of noise absorption owing to vibration, which is characteristic of lightweight, fine powders. Therefore, this method is not applicable to heavy powders with large grain sizes, as their sound absorption predominantly arises from boundary layer viscosity effects.

**Figure 14:**
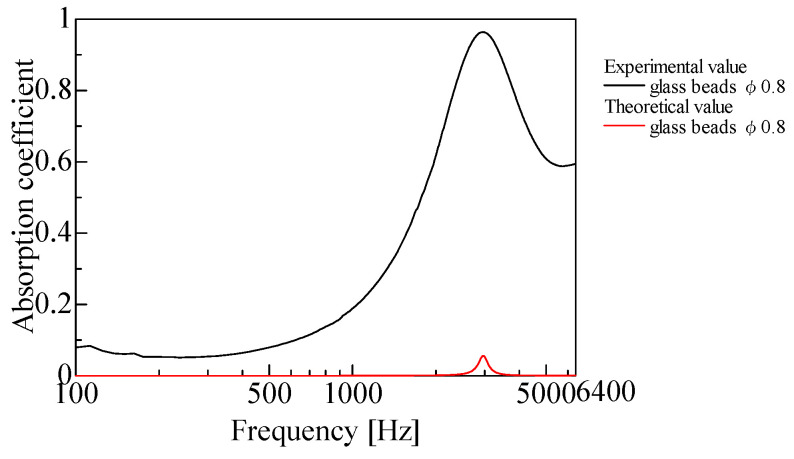
Comparison between theoretical and experimental results for solid glass beads (diameter: 0.8 mm; bulk density: 1.55 g/cm^3^).

As an illustrative example, Figure 14 compares theoretical predictions from this model with experimental results for solid glass beads with a diameter of 0.8 mm and a bulk density of 1.55 g/cm^3^. As shown in Figure 14, the model does not account for sound absorption due to boundary viscosity [5], which limits its applicability to larger and denser powders, such as solid glass beads.

## 5. Conclusions

To estimate the SAC when the powder layers were laminated with various sound-absorbing structures, including a back-air layer, the powder layers were modeled as a continuum, and the transfer matrix was derived and validated. As a result, the following conclusions were reached:

(1) The powder layer was modeled as a 1D beam vibrating longitudinally. The transfer matrix was derived by analogy with an acoustic structure with the same governing equations.

(2) The theoretical values of the SAC of the powder layer alone were calculated using the transfer matrix obtained above and compared with the experimental values, which were close to each other.

(3) A comparison was made between the experimental and theoretical values for the case where the powder layer had a back-air layer. As a result, a shift in the peak sound absorption frequency to a lower frequency caused by adding a back air layer was confirmed in the theoretical and experimental values. 

## Figures and Tables

**Table 1:** Materials used in the experiments.

	Bulk Density *ρ* [g/cm^3^]	First Peak Frequency $f_{p1}$ [Hz]	Feret Diameter [μm]
Granulated silica (Featherfield Co., Ltd., Hiroshima, Japan)	0.0568	550	43
Hollow plastic beads A (Expancel^®^ 920DE40d30) (Japan Fillite Co., Ltd., Osaka, Japan)	0.0298	1100	42
Hollow plastic beads B (matsumoto micro sphere^®^ F–80DE) (Matsumoto Yushi-Seiyaku Co., Ltd., Osaka, Japan)	0.0139	1837.5	110
Hollow glass beads (Hobbico, Inc., Champaign, IL, USA)	0.0800	950	29
Calcium-carbonate-coated hollow plastic beads A (EMC–40(B)) (Japan Fillite Co., Ltd., Osaka, Japan)	0.082	850	53
Calcium-carbonate-coated hollow plastic beads B (EMC–80(B)) (Japan Fillite Co., Ltd., Osaka, Japan)	0.0707	1012.5	70
Calcium-carbonate-coated hollow plastic beads C (EMC–120(α)) (Japan Fillite Co., Ltd., Osaka, Japan)	0.0628	1137.5	120

**Table 2:** Peak SACs and loss factors.

	Experimental Value of the Absorption Coefficient at First Peak Frequency	$\eta_{1}$	$\eta_{2}$
Granulated silica	0.991	0.176	0.257
Hollow plastic beads A (Expancel^®^ 920DE40d30)	0.989	0.164	0.250
Hollow plastic beads B (Matsumoto micro sphere^®^ F–80DE)	1.000	0.262	
Hollow glass beads	0.808	0.034	0.224
Calcium-carbonate-coated hollow plastic beads A (EMC–40(B))	0.996	0.083	0.107
Calcium-carbonate-coated hollow plastic beads B (EMC–80(B))	0.988	0.074	0.115
Calcium-carbonate-coated hollow plastic beads C (EMC–120(α))	0.919	0.051	0.166

**Table 3:** Comparison of the experimental and theoretical values of $f_{p1}$ (powder layer only).

	$f_{p1}$ (Experimental) [Hz]	$f_{p1}$ (Theoretical) [Hz]	Percent Error [%]
Granulated silica	550	555	0.90
Hollow plastic beads A (Expancel^®^ 920DE40d30)	1100	1107.5	0.68
Hollow plastic beads B (Matsumoto micro sphere^®^ F–80DE)	1837.5	1852.5	0.81
Hollow glass beads	950	955	0.52
Calcium-carbonate-coated hollow plastic beads A (EMC–40(B))	850	850	0.00
Calcium-carbonate-coated hollow plastic beads B (EMC–80(B))	1012.5	1012.5	0.00
Calcium-carbonate-coated hollow plastic beads C (EMC–120(α))	1137.5	1142.5	0.44

**Table 4:** Comparison of experimental and theoretical values of $f_{p1}$ (powder layer + nonwoven + back air space).

	$f_{p1}$ (Experimental) [Hz]	$f_{p1}$ (Theoretical) [Hz]	Percent Error [%]
Granulated silica	375	330	12.0
Hollow plastic beads A (Expancel^®^ 920DE40d30)	512.5	502.5	1.95
Hollow plastic beads B (Matsumoto micro sphere^®^ F–80DE)	775	755	2.58
Hollow glass beads	387.5	320	17.4
Calcium-carbonate-coated hollow plastic beads A (EMC–40(B))	362.5	312.5	13.8
Calcium-carbonate-coated hollow plastic beads B (EMC–80(B))	387.5	340	12.3
Calcium-carbonate-coated hollow plastic beads C (EMC–120(α))	400	365	8.75

## Data Availability

The original contributions presented in this study are included in this article; further inquiries can be directed to the corresponding author.

## Abbreviations

SAC	Sound absorption coefficient
SAM	Sound-absorbing materials
5-DOF	Five degrees of freedom

## References

[1] Okudaira Y., Kurihara Y., Ando H., Satoh M., Miyanami K. (1993). Sound absorption measurements for evaluating dynamic physical properties of a powder bed. Powder Technol..

[2] Falegnami A., Tomassi A., Gunella C., Amalfitano S., Corbelli G., Armonaite K., Fornaro C., Giorgi L., Pollini A., Caforio A. (2024). Defining conceptual artefacts to manage and design simplicities in complex adaptive systems. Heliyon.

[3] Okudaira Y., Kurihara Y., Ando H., Satoh M., Miyanami K. (1995). Sound Absorption Characteristics of Powder Beds Comprised of Binary Powder Mixtures. J. Jpn. Soc. Powder Powder Metall..

[4] Sakamoto S., Yamaguchi K., Ii K., Takakura R., Nakamura Y., Suzuki R. (2019). Theoretical and experiment analysis on the sound absorption characteristics of a layer of fine lightweight powder. J. Acoust. Soc. Am..

[5] Sakamoto S., Takakura R., Suzuki R., Katayama I., Saito R., Suzuki K. (2021). Theoretical and experimental analyses of acoustic characteristics of fine-grain powder considering longitudinal vibration and boundary layer viscosity. J. Acoust. Soc. Am..

[6] Sakamoto S., Saito R., Jindai K., Ikeda K. (2024). Conditions for sound absorption caused by longitudinal vibration of lightweight powder. Noise Control Eng. J..

[7] Sakamoto S., Jindai K., Ikeda K., Kawakami Y., Soeta H. (2024). Predicting Lightweight Powders with Useful Sound Absorption Characteristics from Their Specifications. Appl. Sci..

[8] Palacio O., Malfait W.J., Michel S., Barbezat M., Mazrouei-Sebdani Z. (2023). Vibration and structure-borne sound isolation properties of silica aerogels. Constr. Build. Mater..

[9] Biot M.A. (1956). Theory of Propagation of Elastic Waves in a Fluid-Saturated Porous Solid. I. Low-Frequency Range. J. Acoust. Soc. Am..

[10] Tsuruha T., Yamada Y., Otani M., Takano Y. (2020). Effect of casing on sound absorption characteristics of fine spherical granular material. J. Acoust. Soc. Am..

[11] Sakamoto S., Ikeda K., Kawakami Y., Soeta H. (2025). Estimation of transfer matrix by applying the theoretical acoustic incident impedance of a lightweight powder layer to the two-thickness method. Noise Control Eng. J..

[12] Saiki S., Matsumura T., Fujise A. (2012). Speaker System. European Patent.

[13] (2023). Acoustics—Determination of Acoustic Properties in Impedance Tubes—Part 2: Two-Microphone Technique for Normal Sound Absorption Coefficient and Normal Surface Impedance.

[14] Merkus H.G. (2019). Particle Size Measurements: Fundamentals, Practice, Quality.

[15] Lahe A., Braunbrück A., Klauson A. (2020). Modified transfer matrix method for steady-state forced vibration: A system of bar elements. Proc. Est. Acad. Sci..

[16] Ceauşu V., Craifaleanu A., Dragomirescu C. (2010). Transfer matrix method for forced vibrations of bars. UPB Sci. Bull. Ser. D Mech. Eng..

[17] Sugi C. (1964). Resonant Frequencies and Equivalent Mass Coefficients of Longitudinally Vibrating Composite Bars.

[18] Laganà F., Prattico D., De Carlo D., Oliva G., Pullano S.A., Calcagno S. (2024). Engineering Biomedical Problems to Detect Carcinomas: A Tomographic Impedance Approach. Eng.

[19] Delany M.E., Bazley E.N. (1970). Acoustic Properties of Fibrous Absorbent Materials. Appl. Acoust..

[20] Miki Y. (1990). Acoustical properties of porous materials—Modifications of Delany-Bazley models. J. Acoust. Soc. Jpn..

[21] Komatsu T. (2008). Improvement of the Delany-Bazley and Miki models for fibrous sound-absorbing materials. Acoust. Sci. Technol..

